# Dynamically encoded reactivity of Ras enzymes: opening new frontiers for drug discovery

**DOI:** 10.1007/s10555-020-09917-3

**Published:** 2020-08-20

**Authors:** Gyula Pálfy, Dóra K. Menyhárd, András Perczel

**Affiliations:** 1 Laboratory of Structural Chemistry and Biology, Institute of Chemistry, ELTE, Eötvös Loránd University, Pázmány Péter sétány 1/A, 1117 Budapest, Hungary; 2 Protein Modeling Group HAS-ELTE, Institute of Chemistry, Eötvös Loránd University, P.O.B. 32, Budapest, 1538 Hungary

**Keywords:** Protein structure, Ras, Internal dynamics, Conformational ensemble, Dynamics-structure-activity relationship, Conformational selection mechanism

## Abstract

Decoding molecular flexibility in order to understand and predict biological processes—applying the principles of dynamic-structure-activity relationships (DSAR)—becomes a necessity when attempting to design selective and specific inhibitors of a protein that has overlapping interaction surfaces with its upstream and downstream partners along its signaling cascade. Ras proteins are molecular switches that meet this definition perfectly. The close-lying P-loop and the highly flexible switch I and switch II regions are the site of nucleotide-, assisting-, and effector-protein binding. Oncogenic mutations that also appear in this region do not cause easily characterized overall structural changes, due partly to the inherent conformational heterogeneity and pliability of these segments. In this review, we present an overview of the results obtained using approaches targeting Ras dynamics, such as nuclear magnetic resonance (NMR) measurements and experiment-based modeling calculations (mostly molecular dynamics (MD) simulations). These methodologies were successfully used to decipher the mutant- and isoform-specific nature of certain transient states, far-lying allosteric sites, and the internal interaction networks, as well as the interconnectivity of the catalytic and membrane-binding regions. This opens new therapeutic potential: the discovered interaction hotspots present hitherto not targeted, selective sites for drug design efforts in diverse locations of the protein matrix.

## Introduction

A typical protein comprises a few hundred of amino acid residues and thousands of atoms and is folded into a globula of the size of nanoparticles (10–30 nm) with the covalently bound atoms set only 0.1–0.2 nm apart. Objects of any size-range are only perceptible using a light of similar wavelength, which singles out X-ray crystallography (typically utilizing radiation of λ = 0.08–0.15 nm) as the primary source of structural information on proteins carrying atomistic detail. But since crystallography reports the solid-phase structure, for a while, under the strong influence of the impressive array of X-ray-determined macromolecular structures, the inherent dynamic nature of these macromolecules was overlooked. Parallel with the recognition of the intrinsically disordered protein family, the key biological role of highly mobile protein segments has been re-established. The universally used model for the interconnectedness of bioactivity and proteins’ 3D structure (SAR: structure-activity relationship) was thus rephrased to fit for sequences presenting mobile backbone structures, introducing the concept of dynamic-structure-activity relationships (DSAR) [1], especially in case of proteins that have overlapping interfaces with upstream and downstream partners, in case of which even the catalytically distinguished states are better thought of as an ensemble of competing, fluctuating conformers in dynamic equilibrium. Deciphering the biological role and function of proteins, without targeting their dynamical behavior beside their 3D-structural properties, no longer seems possible [2].

### Ras proteins: structure, dynamics, and function

**Fig. 1:**
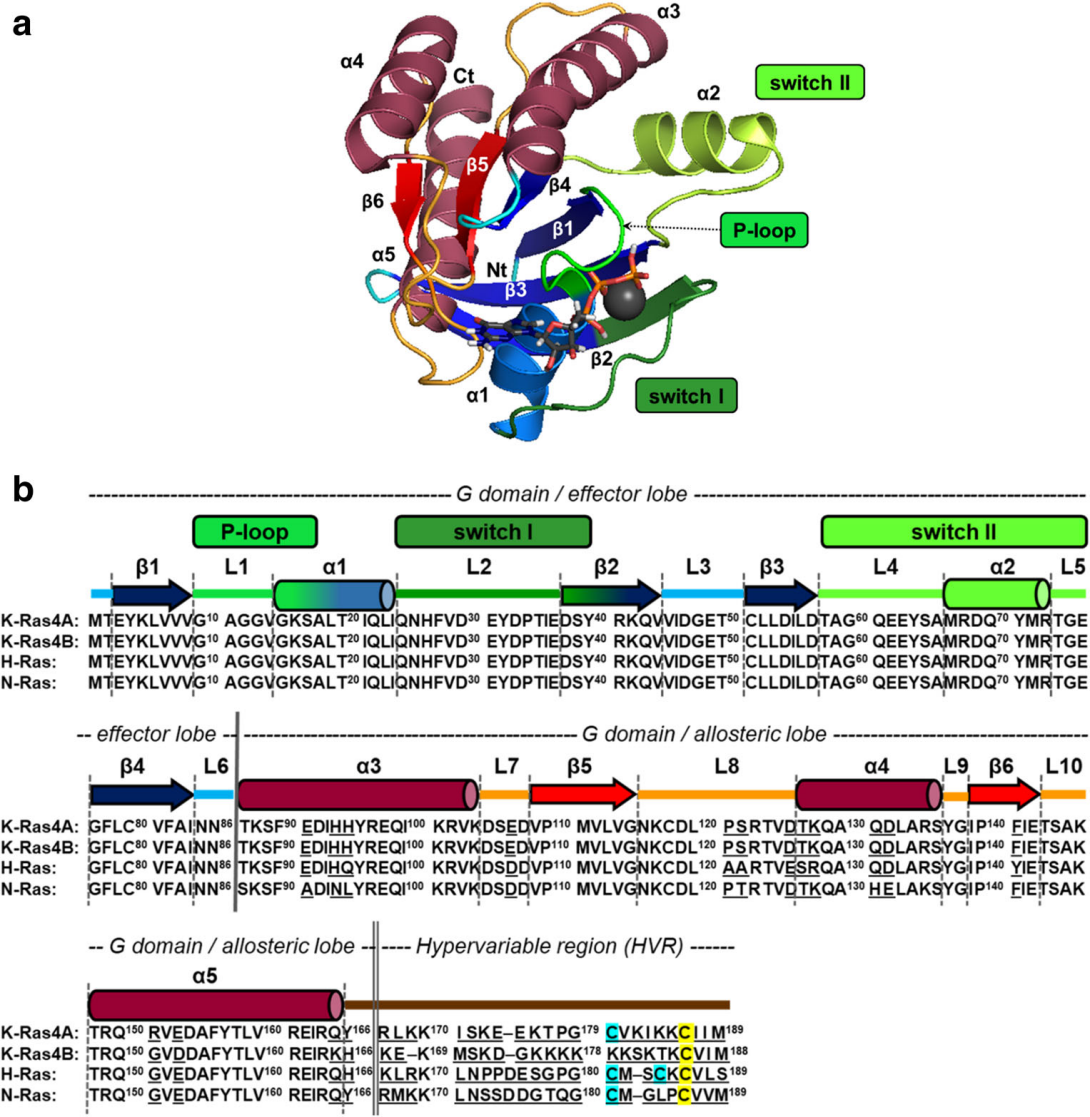
**A** The main structural regions in depicted on 3D structure of G domain of K-Ras-GTP (based on PDB structure: 6MNX, chain **B**). The picture was produced by using The PyMOL Molecular Graphics System, version 2.0 Schrödinger, LLC. software. α1–α5 helices, β1–β6 sheets as well as P-loop, switch I and switch II are indicated. N-terminus and C-terminus are shown as Nt and Ct, respectively, Mg^2+^ is depicted as a gray sphere. **B** Sequence alignment of the four most relevant Ras proteins: K-Ras4A, K-Ras4B, H-Ras, and N-Ras. Residues that are distinct among their sequence are underlined. Secondary structural elements, as well as the P-loop, switch I, and switch II regions are indicated above the sequence colored as in **A**. G domain (effector and allosteric lobes) and hypervariable region (HVR) are shown as well. Farnesylated and palmitoylated cysteines are shown with yellow and cyan background in the sequence, respectively

Kirsten Ras (K-Ras), named after Werner H. Kristen [3], is one of the most frequently mutated oncoproteins in human cancers: it is mutated in 20–30% of all human cancers and often found in colon, pancreatic, and lung cancers [4, 5]. It acts as a molecular switch–regulating pathways associated with cell growth and proliferation [6]. K-Ras, a member of the Ras subfamily small GTPases together with H-Ras and N-Ras among others, is translated in two alternative splice products of K-RAS gene, K-Ras4A and K-Ras4B [7]. Ras proteins are highly homologous, membrane-localized GTPases comprising a guanosine nucleotide-binding domain at the N-terminus (G domain or catalytic domain consisting of 1–166 residues with size of ~ 20 kDa) and a short hypervariable region at the C-terminus (HVR, 167–188/189 residues) [7]. The G domain consists of a Rossmann-fold–like structure, containing 6 β-strands, 5 α helices and the 10 loops between them, can be divided into two lobes: the effector (residues 1–86) and the allosteric lobe (residues 87–166) (Fig. 1A). The effector lobe communicates with the downstream partners, while the allosteric lobe is extended toward the membrane and ends in the membrane-associated HVR segment, which anchors the protein into the plasma membrane after prenylation (or farnesylation or palmitoylation) [8]. The main differences among Ras proteins are found in HVR (with 8% sequence similarity among K-Ras4A, K-Ras4B, H-Ras, and N-Ras), while the effector lobes are completely identical [7] (Fig. 1B).

The effector and nucleotide-binding sites of the effector lobe partially overlap, located on opposing sides of the segment named switch I (residues 25–40). Further regions participating in hosting the nucleotide are the phosphate-binding P-loop (residues 10–17) and switch II (residues 57–76) of the effector lobe [7, 9] and the nucleobase binding- loops of the allosteric lobe (residues 116–120 and 145–147) [7]. Thus, aside from the short and rather rigid P-loop, the catalytic site of the enzyme is primarily formed by flexible segments that—through their shared association with the nucleotide—also create a connection between the lobes. The P-loop is also quite significant from another perspective: 80% of all K-Ras oncogenic mutations occur in the Gly12 position of this segment [4], where the three major mutations are Gly12Cys, Gly12Asp, and Gly12Val (the occurrence is 16%, 40%, and 26% of all G12 mutations). Since K-Ras is the most frequently mutated protein in cancers among Ras superfamily members, this P-loop site carries primary importance.

Ras proteins function as molecular switches in epidermal growth factor (EGF)-dependent receptor tyrosine kinase signal pathways alternating between GDP-bound inactive and GTP-bound active states[10]. In the GTP-bound active form, they can interact with several effector molecules including Raf, PI3K, Tiam1, RalGDS, and PLCs [11]. Although Ras proteins possess both intrinsic nucleotide exchange capacity and GTPase activity, the Ras cycle is controlled and assisted by guanine nucleotide exchange factors (GEFs) and GTPase-activating proteins (GAPs). GEF proteins, such as Sos, facilitate the exchange of the nucleotide substrate, GDP to GTP, resulting in the activation of Ras. GAPs inactivate Ras proteins by significantly enhancing the rate of GTP hydrolysis [7]. Ras oncogenic mutations inhibit the interaction with GAPs and often cause a decrease in the efficiency of intrinsic GTP hydrolysis as well, extending the lifetime of active Ras-bound form. Intriguingly, throughout these numerous interactions, the Ras core structure is not substantially altered; fitting Ras to the various functions is predominantly carried out by the flexibility and communication encoded in a few segments of the protein, creating multicolored dynamics, and also a vehicle for allosteric regulation [12].

### Protein backbone dynamics: a tool to understand biological function

The catalytically significant loops of Ras were “optimized during evolution” to drive protein-protein contacts and sensitively reflect changes in those associations. Thus, in the absence of their physiological partner proteins, they are also prone to initiate self-association. No wonder that in crystal structures and even in more concentrated solutions, a great variety of dimeric and oligomeric states appear that are not necessarily sampled under physiological conditions. These oligomerization processes also distort the switch regions to various extents, thus making it a challenging task to differentiate structural changes of functional relevance from those, merely the side-effect of the applied technology.

To provide additional information pertaining to flexible regions, their solution state or better yet their in-cell conformations should be investigated. These can be targeted by using various nuclear magnetic resonance (NMR) methods. Solution-state NMR, of course, also presents an approximation of the physiological state: the simplified molecular environment of a few ions in a buffer may raise questions about relevance, but the structural information thus derived was shown to be rather useful on numerous occasions. Although *in-cell* NMR method is possible, and thus in principal, protein dynamics could be studied in molecular crowding, applicability is limited to a few special cases due to the too long acquisition time required. Therefore, the currently available experimental data about Ras dynamics mostly originates from *in vitro* NMR dynamical measurements, augmenting the vast amount of structural information carried by the crystal structures of the same systems. Even when studying proteins in the crystal phase, an “imprint” of the inherent internal dynamics remains and can be captured. It is represented by the various differing crystal forms and the differences of the corresponding protein structures (caused by often minute differences in crystallization conditions) and is also manifested in terms of B-factors that indicate the conformational freedom of each atom or group, even within the crystal. In addition, commonly used low-resolution physicochemical methods, ECD, VCD, IR, FRET, DLS, SAXS *etc.*, reflect results which depend on backbone motions.

Another possibility that has opened up considerably in recent years for the study of dynamics is the application of theoretical approaches. Conformational changes that involve the concerted movement of the entire protein on μs-ms time scale can only be studied using molecular modeling methods that rely on force-fields for the description of conformational energy, instead of wave-function and Hamiltonian-based *ab initio* methods. The most popular tool of the field, molecular dynamics (MD) simulations, involves the integration of Newton’s law of motion for the atoms of the system (protein, nucleotide, solvent, membrane *etc.*) approximating the potential energy of each arrangement using classical pair-potentials and simple descriptions of non-bonded interactions (Coulomb’s law and Lennard-Jones-like potentials). Some experimentalist’s skepticism about *in silico* methods, regardless whether the technique incorporates or ignores experimental data, is still present. Curiously, neither NMR nor X-ray raw data can provide a high-resolution picture of any macromolecular structure without *in silico* aid, both applying force field–based calculation steps in reaching the final 3D results. Furthermore, as NMR-derived dynamic properties compare well with MD results, synergy rather than competition characterizes the link between these two methods in describing protein internal motions, as presented in this review paper.

Proteins of merely tenth of nanometers (10^−9^ m) in size [13] experience fluctuating motions on a large time scale, spanning about 18 orders of magnitude: 10^−15^–10^3^ s [14–16]. Enzyme-catalyzed bond cleavage and formation, as well as electron, proton, hydride- and methanide-ion transfer *etc.*—the chemistry of proteins—occurs on a fs → ps (10^−15^ → 10^−12^ s) time scale. Side chain rotation and loop-restructuring motions span the adjacent time regime, ps → ns (10^−9^ s), whereas the upper limit of the range is about the time required for an average protein to randomly tumble in water (5–20 ns) under physiological conditions. Molecular tumbling is used as the reference motion (see below) for macromolecules. The time needed for 3D-folding strongly depends on the nature of the protein and can be as fast as just a few nanoseconds, or slower (up to seconds).

NMR can report, by using special pulse sequences (T_1_, T_2_, steady-state heteronuclear ^1^H, ^15^N NOE, RDC, CPMG, CEST, EXSY, *etc.*), explicit data on all of these different time scales of motions. As a result, for a “Ras-sized” protein comprising 150–200 amino acids, quantitative and residue-specific data on backbone dynamics, conformational equilibria, exchange between conformers, 3D-fold formation, ligand- and receptor-binding *etc.* can routinely be obtained [17, 18].

The rotational correlation time, *τ*_c_, used as the reference of molecular motion (tumbling in solution) is the time needed for a protein to rotate one radian. Thus, *τ*_c_, depends on the size, shape, plasticity, *etc.* of the macromolecule, plus on its molecular environment defined by solvent viscosity, pH, temperature, *etc.* In a diluted solution at physiological conditions, *τ*_c_ is on the order 10–20 ns for proteins of moderate size. For example, a GTP analogue–bound Ras protein has a *τ*_c_ ~ 11 ns, but binding to any of its protein partners (GEF, GAP, effectors) and/or forming transient complexes increases *τ*_c_ significantly [19]. With the introduction of the internal correlation time, *τ*_e_ referring to much faster motions than the reference tumbling, a protein’s motion can be described by the sum of slower, global (*τ*_c_) and faster, local (*τ*_e_) motion(s) [20]:

1 $$ {\tau}^{-1}={\tau_{\mathrm{c}}}^{-1}+{\tau_{\mathrm{e}}}^{-1} $$

where the *τ* correlation time characterizes the overall motion.

Excited nuclear spins (e.g., ^1^H, ^13^C, ^15^N) during an NMR experiment relax by interacting with the fluctuating random electromagnetic field generated by the stochastic rotational diffusion of other molecules. This is reflected by the decline of the NMR signal detected by both *T*_1_, responsible for the loss of signal intensity, and *T*_2_, manifesting in terms of signal’s broadening [21]. The spectral density function, *J*(*ω*), quantifies the strength of the fluctuating electromagnetic field at an *ω* (= 2πυ) frequency. If correlation between some NMR is measurable, *T*_1_ = *R*_1_^−1^, *T*_2_ = *R*_2_^−1^, steady-state ^1^H-^15^N HetNOE, and *J*(*ω*) is established *via* a suitable model, then the complex motion (fast and slow) of a macromolecule can be explicitly described. Molecular motion, the origin of *J*(*ω*), is characterized by the autocorrelation function *C*(*t*). *C*(*t*)  that describes the probability of a vector (*e.g.* a chemical bond, for example that of the ^1^H-^15^N) to be oriented similarly with respect to the external magnetic field after time *t*. In other words, *C*(*t*) gives the “memory” of how long a molecular conformation remains “unchanged.”

“Fast dynamics” refers to the 100 ps → ns internal motions. Data describing such motions can be acquired on non-selectively isotopically labeled (^15^N, ^13^C) proteins. The different relaxation mechanisms such as dipole-dipole (DD) and chemical shift anisotropy (CSA), associated with ^1^H-^15^N and ^1^H-^13^C, were established [22]. In fact, DD-relaxation measured by the different ^1^H-^1^H NOESY experiments form the basis of quantitative structure determination by NMR, by assuming that the relaxation intensity (efficacy) of spatially close protons is proportional to their distance (~ 1/r^6^), where *r* is the distance between them. The Lipari-Szabo, or model-free approach (LS-approach), perhaps the most widely used model, describes a globular protein’s rotational correlation time, *τ*_c_. LS-approach assumes that (*i*) the internal motion characterized by *τ*_e_ (≤ 0.01–0.1 ns) is at least two magnitudes faster than *τ*_c_ (≥ 2–3 ns) [23, 24] and (*ii*) that *τ*_c_ and *τ*_e_ are uncorrelated, which is mostly typical for globular, compact, and well-structured proteins. In this case, the autocorrelation function, *C*(*t*), can be subdivided as follows:

2 $$ C(t)=C{(t)}_{\mathrm{global}}\cdotp C{(t)}_{\mathrm{internal}} $$

Furthermore, in this case, the explicit form of *C*(*t*) can be written as

3 $$ C(t)=0.2\ \left[{S}^2{\mathrm{e}}^{-t/\tau \mathrm{c}}+\left(1\hbox{--} {S}^2\right)\ {\mathrm{e}}^{-t/\tau \mathrm{e}}\right] $$

where the order parameter *S*^*2*^ (0 ≤ *S* ^*2*^ ≤ 1) reflects on the extent of motional restriction. A value of *S*^2^ close to 1.0 indicates a rigid, while that close to 0.0 signals a highly dynamic, internal motion with respect to the reference rotational diffusion (tumbling of the protein). An alternative of the LS-method is the reduced spectral density mapping approach [25], relying directly on measured data (*R*_1_, *R*_2_, and steady-state ^1^H-^15^N HetNOE values) is a semi-quantitative approach for describing fast dynamics in terms of *J*(0) (value of spectral density at zero frequency, when *ω* = 2π*υ* = 0 Hz), *J*(*ω*_N_) (spectral density at nitrogen Larmor frequency) and *J*(0.87*ω*_H_) (spectral density at 0.87 times proton Larmor frequency). As 0, *ω*_N_, and *ω*_H_ are distinct, *J*(0), *J*(*ω*_N_), and *J*(0.87*ω*_H_) are good descriptors of slow, intermediate, and fast backbone dynamics, respectively. The most common plot in use is that of *J*(*ω*_N_) as function of *J*(0) showing a residue-specific mapping of backbone motions.

“Slow dynamics”  refers to the 300-μs to 10-ms internal motion regime of a protein, often called the conformational exchange regime of motions; *τ*_ex_ is measured by CPMG-T_2_ relaxation dispersion experiment, a “variant” of the common spin-echo NMR. The incremented echo-delays here probe exactly the time range in question (100 μs → 10 ms) and acquire changes in terms of *R*_2,obs_ = *T*_2,obs_^−1^ [26, 27]. The goal of these measurements is to distinguish the intrinsic line-width measured by *R*_2,0_ = *T*_2_^−1^, from resonance line broadening arising from exchange between different (conformational) states, *R*_ex_:

4 $$ {R}_{2,\mathrm{obs}}={R}_{2,0}+{R}_{\mathrm{ex}} $$

Thus, the extent of exchange associated with transitions between conformational states of interest can quantitatively be characterized [28]. Slower time scale motions are to depict and decipher conformationally distinct states of proteins, in other words thermal fluctuation–induced large structural changes. As conformers are indeed diverse, substantial chemical shift differences, Δ*δ*, are typically associated with these protein states. Slower chemical exchange, *τ*_ex_ ≈ 3–30 ms, can be measured by CEST and DEST NMR experiments [29].

Various biologically relevant events (ligand binding, folding ↔ unfolding, catalysis, *etc.*) are coupled to backbone, side chain, domain, and loop motions occurring at an exchange rate characterized by the exchange rate constant *k*_ex_ of 1/s < *k*_ex_ < 10^5^/s. The term *k*_ex_ is defined as *k*_ex_ *= k*_on_ + *k*_off_ for a two-state model for the exchanging species. Exchange affects the fundamental NMR measurables such as chemical shift, resonance linewidth, and signal intensity. Resonance broadening of selected residues can be such that the NMR signal completely vanishes, observed for proteins *via* the “disappearance” of HSQC signals. Two- or three-state exchange can numerically be handled relatively easily, thus distinguishing slow (*k*_ex_ << │Δ*υ*│), intermediate (*k*_ex_ ≈ │Δ*υ*│) and fast (*k*_ex_ >> │Δ*υ*│) exchange, where Δ*υ* is the difference between the exchange of *υ*_a_ and *υ*_b_ resonances (in Hertz) of population *p*_0_ (ground state, with large population) and *p*_E_ (excited state with low population) in the two-state model.

In crystallography, B-factors stand for the uncertainty in the coordinate values caused primarily by thermal fluctuation of the atoms within the crystal. When high resolution can be achieved, even the anisotropy of such thermal fluctuations can be denoted. However, this kind of dynamics “added” on to a static structure does not reflect the true complexity of backbone and side chain internal motions of proteins. On the other hand, in case of NMR measurements—reflecting on a broad range of characteristic and biologically relevant motions—it is impossible to obtain information on all the atoms. Thus, only motions of selected atoms (e.g., ^1^H-^15^N) can be quantitatively characterized.

**Fig. 2:**
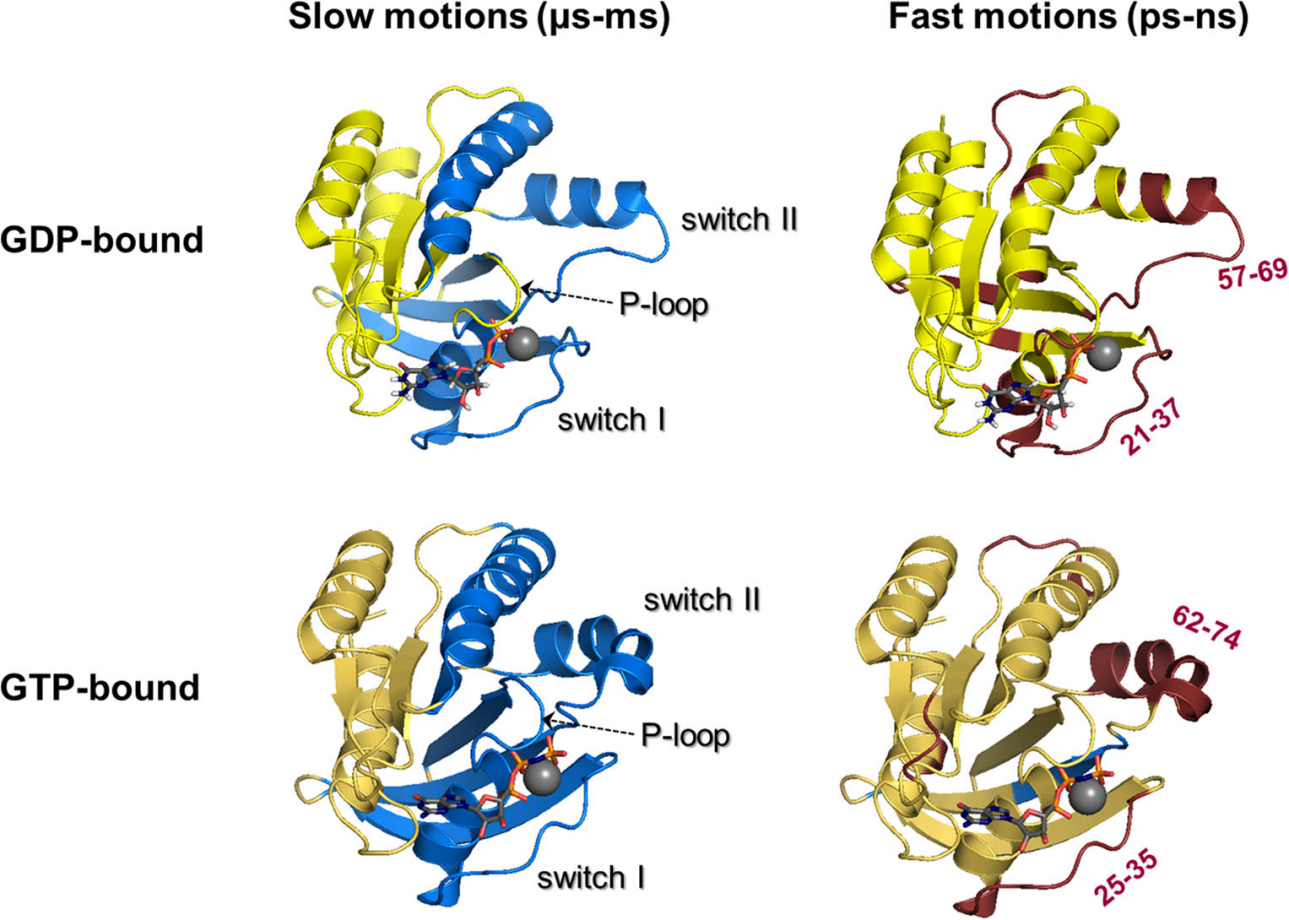
Summary of the results of NMR dynamics studies for Ras proteins: regions with slow and fast time scale motions of H-Ras in GDP- and GTP/GTPγS-bound states (upper and bottom panels, respectively). Slow motions (left hand side, blue-colored) are μs-ns time scale, conformational motions, rigid regions, slower than the average tumbling motions characterized by rotational correlational time: *τ*_c_, based on the results of Mao et al. [30] (GDP-bound) and Chen et al. [31] (GTP-bound). Fast motions (right hand side, red colored) are ps-ns time scale, internal fast motions, flexible regions, faster than the average tumbling motions characterized by rotational correlational time: *τ*_c_, based on the results of Thapar et al. (Thapar 2004) (GDP-bound) and Vo et al. [19] (GTPγS-bound, no literature for native GTP-bound form). The active regions are found in Table 1. The pictures were produced by using The PyMOL Molecular Graphics System, version 2.0 Schrödinger, LLC software, using PDB codes: 4OBE (H-Ras-GDP) and 3KY8 (H-Ras-GppNHp) PDB structures

The dynamics of Ras proteins were studied experimentally by NMR using two different approaches: based on the bound nucleotide (^31^P-NMR, using labeled nucleotide in presence of the unlabeled protein) or the protein itself: NMR methods for probing protein dynamics in fast and slow time scales, using isotopically labeled protein saples. Both approaches were extensively applied for H-Ras, whereas only a few studies were conducted for K-Ras or other Ras family members (the most recent results are summarized in Fig. 2 and Table 1 for H-Ras in GDP- and GTP-bound forms). The most often examined catalytic domains of H-Ras and K-Ras have not only a sequence identity of 93.5%, but also their similar dynamic properties were demonstrated by nucleotide-based ^31^P-NMR [33], and protein-based slow dynamics studies using CPMG measurements for both proteins bound to a non-hydrolyzable GTP analogue, GppNHp [34]. Moreover, the structure of other small G-proteins such as Rap, Ran, Rho, Rab *etc.* as well as G_α_-subunit of heterotrimer G-proteins, which also play central role in signal transduction cascades, is highly homologous to Ras proteins [35], suggesting that Ras proteins can be a proper model for understanding the dynamics encoded in their structure.

**Table 1:** Summary of the results of the most recent NMR dynamics studies for Ras proteins: regions with slow and fast time scale motions of H-Ras in GDP- and GTP/GTPγS-bound states. No results for native GTP-bound H-Ras are found in literature for fast time scale motions; thus, analogue using experiments are shown in that case. Experimental details: GDP-bound H-Ras: H-Ras(1-171)-GDP, pH 7.5, 278 K, RD/CPMG measurements (slow dynamics) [30] and H-Ras(1-166)-GDP, pH 6.5, 298 K, T_1_, T_2_, ^1^H,^15^N-HetNOE measurements (fast dynamics) [32]; GTP-bound H-Ras: H-Ras(1-171)-GTP, pH 7.6, 288 K, CPMG + CEST measurements (slow dynamics) [31], H-Ras(1-166)-GTPγS, pH = 7.5, 290 K, T_1_, T_2_, ^1^H,^15^N-HetNOE measurements (fast dynamics) [19]

		Slow motions (μs-ms)	Fast motions (ps-ns)
GDP-bound	Active regions/residues	β1, α1, L2 (switch I), β2, β3, L4, α2 (switch II), α3 [30]	13–15 (P-loop), 21–37 (switch I), 43, 47–51, 57–69, 74 (switch II), 89, 94, 106–109 residues are flexible [32]
GTP-bound	Active regions/residues	β1, L1 (P-loop), α1, L2 (switch I), β2, β3, L4, α2 (switch II), β4, α3 [31]	residues 25–35 (switch I), 62–74 (switch II), 104–107, 121–123, 156 are flexible residues 49, 54, 56–59 (switch II) are rigid [19]

### The overall Ras conformational ensemble

Based on the crystal structures of various Ras forms, four major conformational states of the protein can be differentiated: GDP-bound, GEF-bound, and two GTP-bound states: those of the inactive state 1 with switch I released from the catalytic site proposed to function as a stable pool of the GTP-loaded form [36] and state 2 that is the catalytically active form, capable of intrinsic GTP hydrolysis and effector binding, and also forming the catalytically enhanced GAP-complex. The conformational heterogeneity of GTP-bound Ras was determined long ago: there were missing signals in the NMR spectra of H-Ras and N-Ras bound to GTP or its analogues, which was interpreted as intermediate chemical exchange that caused signal line broadening [37–42]. Peaks were missing in the P-loop (residues 10–13), switch I (residues 31–39), and in the switch II (residues 57–64 and 71) segments (varied depending on the exact conditions of the experiments and the bound nucleotide or analogue). A number of broadened peaks appeared by change of temperature confirming the presence of intermediate exchange, which was further strengthened by the detection of slow motions for the adjacent regions of undetected peaks in β1 (residues 7, 8), P-loop (residue 14), and switch II (residues 56, 66, 70) of H-Ras-GppNHp–bound form [40]. The addition of Raf resulted in the re-appearance of several missing signals of the free form, suggesting that interaction with the effector shifted the conformational equilibrium [40]. State 1 and state 2 were differentiated by nucleotide-based ^31^P-NMR studies through nucleotide detection which showed the existence of two different conformational states of H-Ras(1-189) when bound to non-hydrolyzable GTP analogue, such as GppNHp representing GTP-bound Ras form [43, 44]. The finding of two distinct sets of signals in the ^31^P-NMR spectrum indicated exchange in slow time scales. Adding the effector Raf shifted the conformational equilibrium toward state 2, showing that this conformation is the active form corresponding to effector binding. Mutation at residue Thr35 (in Thr35Ala and Thr35Ser mutant proteins) was shown to shift the conformational exchange toward state 1, and since these variants are unable to bind effector proteins, this was considered the inactive form of the GTP-bound state. Later, other ^31^P-NMR experiments suggested that state 1 might be similar to the conformation assumed when Ras associates with GEF [45], the protein assisting in nucleotide exchange. The solution-state structure of H-Ras(1-166)-T35S-GppNHp belonging to state 1 was determined as well [41], suggesting that the main difference between the two states is the absence or presence of hydrogen bond between side chain of Thr35 and Mg^2+^. Interestingly, the mobility pattern of this Thr35Ser-GppNHp mutant representing state 1 of Ras-GTP is more similar to Ras-GDP than to the wild-type Ras-GppNHp [41]. State 1 was further characterized by using a nucleotide analogue showing more native dynamical properties (GTPγS). In case of H-Ras-GTPγS, the authors found flexible regions showing fast time scale rapid internal motions at switch I (residues 25–35) and switch II (residues 62–74) of the effector lobe, but also at L7 (residues 104–107), L8 (residues 121–123), and Phe156 of the allosteric lobe. Measurements were also carried out in the presence of GEF, but these results were harder to interpret as they used 2:1 Ras:GEF ratio that gave rise to complex dynamical processes consisting of the transition between GTPγS- and the GEF-bound state of Ras, as well as the intrinsic dynamics of both forms. This was reflected in the results of the Lipari-Szabo model-free analysis of the obtained relaxation data, since free Ras-GTPγS has very few residues active in slow time scale motions characterized by *R*_ex_ (mainly those of residues 49, 54, 56–59 of switch II), while in the presence of GEF, *R*_ex_ shows a completely different landscape, with more implicated segments spread in different regions of the protein: β1 (residue 3), α1 (residues 17, 21), β2 (residue 38), β3 (residues 55, 57), and switch II (residues 62, 70, 71) in the effector lobe and α3 (residues 88, 90), α4 (residues 126, 128, 130), and β6 (residue 146) of the allosteric lobe [19]. In a very recent study, the state 1 ↔ state 2 conformational transition (at 15 °C) of the native GTP-bound form of H-Ras(1-171) was characterized by utilizing a combined CPMG-CEST approach. The authors assigned the lower populated conformational state to state 1 of Ras-GTP by comparing the obtained chemical shift differences for backbone N-atoms with those of Ras-Thr35Ala-GTP in very good agreement. They also found that the residues mostly affected in the state 1 ↔ state 2 slow motions are found in the effector lobe and the α3 helix of the allosteric lobe [31].

H-Ras(1-189)-GDP was also studied by ^31^P-NMR, and it was found that not only wild type but also the Thr35Ala and Thr35Ser mutants have two different states (proved by two different signal sets) exchanging in slow time scale [46]. Protein-based, ^15^N-slow dynamics measurements strengthened this finding, as H-Ras(1-171)-GDP was found to exhibit exchange process in slow time scale [30].

In replica exchange MD simulation (a technique that results in enhanced sampling of the conformational space) of the GTP-bound complex of H-Ras, conformations belonging to the functional state 2 could be further grouped into sub-states based on the position of switch I residue, Tyr32. When Tyr32 reaches toward the γ-phosphate of GTP, the protein matrix is in a conformation that is similar to that seen in Ras-effector complexes, while in the presence of outward-pointing Tyr32, the protein assumes a structure reminiscent of the GAP-bound states [47].

**Fig. 3:**
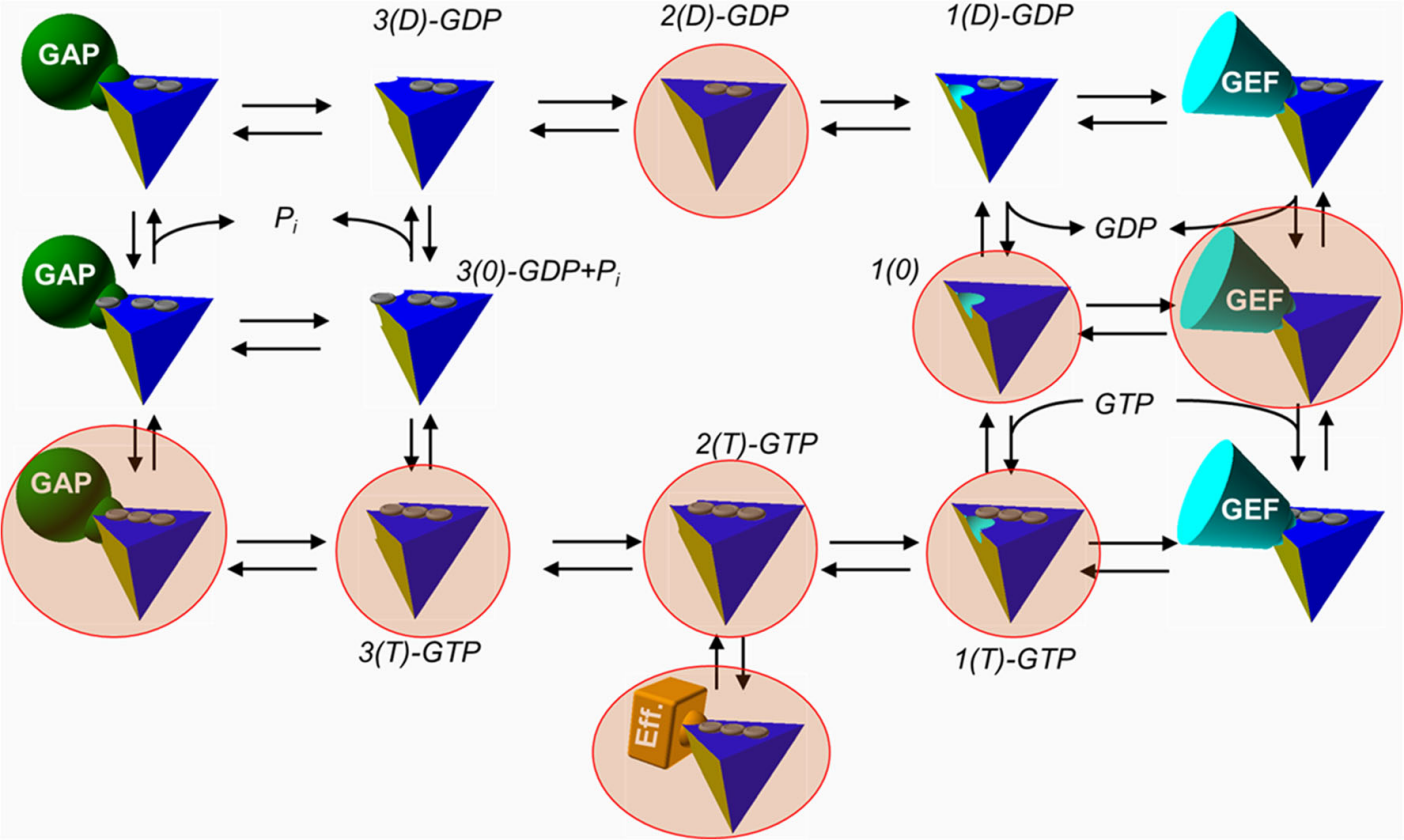
Hypothetical conformational states of Ras proteins in Ras cycle according to Kalbitzer et al. [48]. Those states which were identified and studied experimentally (by NMR or X-ray methods) are encircled and designated by light red background

The most detailed model for the main conformational states interconverting in slow time scale was proposed by Kalbitzer et al. [48]. They introduced a total of 15 states involved in Ras cycle. Three states for the GDP-bound free forms (preformed conformations for GEF-binding (1(D) state), GAP-binding (3(D) state) and the intermediate between them (2(D) state)), as well as the GAP- and GEF-bound states. The same conformations were defined for GTP-bound forms (1(T): ready for GEF-binding, 3(T) ready for GAP-binding, 2(T): intermediate between them and the GEF- and GAP-bound conformations). They also assumed intermediate conformations between GDP- and GTP-loaded states both in GEF/GAP-free and GEF/GAP-bound forms: two of which are nucleotide free forms (connecting GEF-bound GDP- and GTP-loaded conformations, and the same without GEF, which is named as 1(0)), and the other two are intermediate in GTP hydrolysis (GDP + the hydrolyzed inorganic phosphate before product release) either bound to GAP or in the standalone form. The last conformation is the active state, prepared for effector binding which can be directly derived from 2(T) state, a free GTP-bound form. They identified the previously observed state 1 and state 2 conformations of GTP-bound forms as 1(T) and 2(T) states. In their work, they used high-pressure NMR to study the pressure dependence of chemical shifts for backbone-amide N-H signals of H-Ras(1-166) bound to analogue GppNHp and found four different states appeared in this thermodynamic space: 1(0), 1(T), 2(T), and 3(T) based on detailed analysis of the obtained thermodynamic parameters. Although a number of previously presented experimental data confirm the existence of some of these conformational states, not all of them were detected yet (Fig. 3). It should be noted that as a result of the motions in the sub-nanosecond (fast) time scale, more sub-states are superimposed onto each conformational state.

Understanding the interconnectedness of (primarily) the G-domain, and how allosteric regulation is carried out through coupled side chain fluctuations was studied using the conditional time-delayed correlation approach and Gaussian network models analyzing MD simulations of the GDP and GTP-bound forms of K-Ras. It could be deduced that the motion of the P-loop (L1), β4, β5, and β6 is stiffly coupled to the overall motions of the protein while the switch regions show high flexibility. The analysis also indicated that the switch regions are connected, with switch II driving the motion of switch I [49]. Conserved water molecules were also shown to be an integral part of Ras structure and catalysis [50, 51].

In conclusion, it seems that the dynamic conformational ensemble of both the GDP- and GTP-loaded state carries imprints of multiple steps of the catalytic cycle, preparing the system for both signal transduction and switch-off—even before the interaction partners arrive.

### Nucleotide binding and exchange

GTP is a longer and more negatively charged molecule than GDP. Both nucleotides bind to Ras proteins in a conformation that runs parallel to the switch I loop, but GDP does not fill the entire nucleotide-binding cleft; most significantly, it does not reach to the switch II region, which thus remains quite flexible. On the other hand, GTP, elongated by the additional phosphate group, does reach switch II. In fact, its γ-phosphate is clamped between 3 “fingers” of the nucleotide-binding lobe: the tight P-loop (the site of the majority of oncogenic mutations), switch I, and the L4 loop belonging to switch II (carrying the also oncogenic residue, Gln61). Thus, the appearance of the γ-phosphate creates a connection between the flexible and pliable switch regions and the firmly rooted P-loop.

Accordingly, in a MD study that compared the dynamics of H-Ras to representatives of the other two GTPase superfamilies (heterotrimeric G-proteins and protein synthesizing GTPases), a conserved pattern was recognized: the active, GTP-loaded form of all three families displays stronger correlation within the nucleotide-binding lobe and between the two lobes as well, resulting in an overall more rigid structure than in the case of the GDP-bound forms. The authors showed that disrupting inter-lobe interaction hotspots such as the Met72 (switch II)–Val103 (α3) or Asp47/Glu49 (L3)–Arg164 (α5) reduces the correlation between the P-loop and switch I too and thus perturbs the fixing of the active nucleotide [52]. This connection works both ways, structural integrity of the allosteric lobe and its communication with the active site affects GTP binding, just as GTP binding rigidifies the entire protein matrix. This communication establishes a connection between the nucleotide-binding communities and the site of membrane binding since the membrane-penetrating hypervariable region is a direct continuation of α5. Correlated motion of the nucleotide-binding site and the membrane interacting C-terminus was also shown to be a requirement for nucleotide exchange, involving collective motions of the entire protein in a detailed normal mode analysis of accelerated MD simulations, which again underlines the significance of the dynamic linkage between the lobes [53].

Overall stiffening in the GTP-bound state was also found in case of K-Ras, reflected in higher mean spring constant values derived from correlation analysis of MD simulations, especially for switch I (L2), β2, β3, and α3. Correlations were shown to exist between α1-switch I region and L10-α5 segment [49] possibly conveyed by the nucleotide itself that interacts with L10. In this sense, simply the elongation of the GTP nucleotide by the terminal phosphate group, as a rigid body lever, switches on the catalytically essential connection between nucleotide exchange and membrane anchorage. The connection between these far lying sites is physically conveyed by—sometimes just transiently present—H-bonds scattered along the long path [49]. The H-bonded network connecting the catalytic and allosteric regions was also proposed to play a key role in isoform-specific biochemical properties of Ras proteins [54]. The nucleobase of the nucleotide is coordinated by switch I, L8, and L10, of which switch I and L10 are spatially adjacent, while L8—carrying isoform-specific residues—lies on the other side of L10; thus, subtle changes in that region also might influence the crosstalk between switch I and the allosteric lobe. Interestingly, the most redox-sensitive site of Ras proteins, Cys118, is also located on L8. The oxidation of Cys118 (nitrosylation by reactive nitrogen species) affects tumorigenesis *via* influencing the nucleotide exchange rate and intrinsic GTPase activity in an isoform-specific manner [55], illustrating again the very real connection between these distant loci. In another study, local network entropy of the state 1–state 2 transitions of GTP-bound H-Ras based on parallel cascade selection MD simulations was calculated and the transition states of the process described. The key steps here also involved the formation of switch II–α3 interlobal H-bonds [56].

Ras-GTP forms are difficult to study experimentally as the NMR measurements require quite a long time (1–3 days) which is indeed longer than the rate of GTP hydrolysis (hour(s)). Therefore, non-hydrolyzable (or at least very slowly hydrolyzable) nucleotides, such as GppNHp, GppCH_2_p, and GTPγS, have long been utilized for these measurements. The effects of GTP nucleotide analogue on structure and dynamics were investigated first by comparing ^1^H,^15^N-HSQC spectra of H-Ras(1-171) bound to GTP, GTPγS, and GppNHp at 25–30 °C [40]. In all cases, missing peaks were found but their number increased in the order of GTP, GTPγS, and GppNHp suggesting that the rate of conformational interconversion increases respectively. The effect of nucleotide analogues GppNHp, GppCH_2_p, and GTPγS on H-Ras(1-166) or full-length H-Ras(1-189) dynamics were studied on the slow time scale through the nucleotide-based ^31^P-NMR methods, and the similarity of GppNHp and GppCH_2_p as well as their divergence from the behavior of GTP and GTPγS was demonstrated [57–59]. Comparison of the native GTP, GTPγS, and GppNHp in case of H-Ras(1-171) by CEST measurements at 5 °C—in addition to a close relative protein Rheb(1-169) [60]—showed that the distribution profile of *k*_ex_ *vs. p*_e_ of GTPγS mimics the native GTP more efficiently than GppNHp. Moreover, it was proved very recently that GppNHp causes distortion in structure not only dynamics in case of H-Ras-Thr35Ala(1-171) mutant [61].

One solution for the nucleotide analogue problem was provided recently by Chen and coworkers as they added a trace amount of Sos, a GEF protein, to H-Ras(1-171) in 1:1200 ratio and an excess of GTP in the buffer (1:12 of Ras-GTP) resulting in a continuous exchange of GDP formed by GTP hydrolysis of Ras to GTP derived from the solvent [31]. They found a difference from the results of earlier studies performed using the GppNHp analogue with H-Ras(1-166) at 17 °C [62] or K-Ras(1-171) measured at 20 °C [34] indicating the involvement of C-terminal residues (140–160 region) in slow motions: Chen and coworkers found that these regions were not involved in slow motions when using the native GTP nucleotide [31]. These findings thus advise caution when interpreting results obtained using the GppNHp analogue.

We have designed a method to extend the lifetime of Ras-GTP complex by adding excess of GTP into the buffer without addition of Sos or any other protein which can interact with Ras [63]. By this approach based on the enzyme-free nucleotide exchange, we managed to get “unchanging” NMR signals of K-Ras(1-169)-GTP complex for several days enabling us to perform detailed NMR relaxation measurements at 25 °C. The transfer from GDP-bound state to GTP-bound form and back proceeds along the following pathway: 1(D)-GDP → 1(0) → 1(T)-GTP → 2(T)-GTP → 3(T)-GTP → 3(0)-GDP-P_i_ → 3(D)-GDP → 2(D)-GDP → 1(D)-GDP. GTP excess shifts the ratio of the affected reaction rates to stabilize Ras-GTP complex for a longer time. This approach prevents to interpret any possible artifacts arising from the addition of GEF.

### Oncogenic mutations

The sites of the most frequent oncogenic mutations of Ras proteins are the P-loop—a short, well-structured segment located between β1 and α1—and the flexible switch II loop, L4. While this latter, Gln61X-type mutations involve the key residue of GTP hydrolysis, the much more frequent P-loop mutations (Gly12X, Gly13X) simply create a protrusion on the outer surface of the protein. Their primary effect is that they clash with GAP, dislodging it from its intended position, blocking the advance of its “Arg finger” that would provide the boost of enhanced hydrolysis. The unsheltered location of position 12 mutations in itself greatly hinders drug design efforts, and it is a further complication that the overall structure of the protein seems unaffected by their presence. Therefore, to design inhibitors that recognize and selectively bind the mutant proteins, the more subtle differences between the wild-type and mutant systems need to be mapped and targeted. That these subtle differences do exist is reflected in the fact that mutations also alter nucleotide exchange rates and intrinsic hydrolysis to various extents [64]—the mechanism of which is harder to decipher.

A comprehensive MD study of position 12 mutants in their GDP- and GTP-bound forms was carried out recently (encompassing 85 simulations) that demonstrated that the dynamics of residue 12 itself was not substantially altered by the mutations —reflecting the very stable fold of the P-loop, neither were there easily perceived differences between the derived ensembles. However, detailed principal component and Markov state model analysis of the trajectories indicated that conformational distribution of wild type and mutant proteins is subtly different; there are conformational states of the wild-type systems (both GDP- and GTP-bound) that the mutants do not sample and *vice versa*. The liberation of the α4 helix was shown to be the characteristic of the GTP-bound forms of the Gly12Ala, Gly12Asp, Gly12Arg, and Gly12Val variants, while the mobility of switch II was found to be considerably reduced in case of Gly12Arg. The authors also identified a hydrophobic hub network connecting the P-loop and the C-terminal α5 helix (Val14-Met72-Phe78-Leu79-Phe90-Ile100-Val114-Ala146-Ala155-Phe156-Leu159) that existed in all model systems, but with small variances in case of the mutants. For example, the shift of Met72 was detected in the case of Gly12Cys, Gly12Arg, and Gly12Val mutants. Thus, modest—but recognizable—differences were identified in the dynamics and interaction networks of the  wild-type system and those carrying oncogenic mutations [65].

Comparing the activated (GTP-bound) forms of two different isoforms of Ras and their Gly12Asp, Gly12Val, and Gly13Asp variants has shown that while both K-Ras and H-Ras are well-ordered systems with flexible switch regions, the extent of the conformational freedom of the switches is both isoform and mutational-state selective: switch I was most flexible in H-Ras-Gly12Asp and H-Ras-Gly12Val and least flexible in the H-Ras-wild-type and H-Ras-Gly13Asp as well as K-Ras-Gly12Asp [66]. Different interaction networks were identified in H-Ras and K-Ras, and in wt and mutant forms. Interactions of Tyr32 were found to be quite diverse in the different systems, and in case of mutants, enhanced coupling between the switches was detected *via* the Glu37/Asp38–Tyr71 interaction. Other works found the changes in the switch II–α3 interaction issued by the Gly12Asp mutation of K-Ras to be of significance [67] and detected the opening of the nucleotide-binding pocket caused by the increased P-loop–switch II separation [67, 68].

An earlier ^31^P-NMR study suggested that oncogenic Gly12Val mutant of H-Ras in GDP-bound form has only one state meaning the lack of slow time scale motions in contrast to the wt form exhibiting slow exchange by the appearance of two different sets of ^31^P-signals form [46]. This indicated that oncogenic mutation can alter slow time scale motions of Ras in GDP-bound. However in a more recent protein-based study, identical regions were found to be involved in conformational exchange with similar *k*_ex_ value in case of the wild-type and the Gly12Val mutant of GDP-bound H-Ras(1-171) (2910 and 2930 s^−1^ for wild type and mutant, respectively) at 5 °C. However, the population of minor state was estimated to be 24–49% smaller in the Gly12Val variant than in case of the wt indicating a mutation-induced shift in population distribution in the slow time scale motions [30]. We have recently compared K-Ras wild type and its oncogenic Gly12Cys, Gly12Asp, and Gly12Val mutants in their GDP-bound forms by CPMG measurements and found no significant difference in the excited state’s populations [69].

Results demonstrate that seemingly isolated mutations cause far-reaching but subtle differences in the overall structure of Ras proteins and that mutant-specific transient states, allosterically modified sites, and shifts in the internal interaction networks can be distinguished.

### Membrane association

The physiological form of activated, functional Ras proteins is not only GTP-loaded but also carries post-translational modifications which assist in forming a dimeric membrane-bound state, ( although the issue of dimerization is still not unambiguously resolved as quoted by Frank McCormick in their review of 2017 [70]). Ras proteins are anchored to the membrane through their prenylated hypervariable region, the isoform-dependent sequence of which leads to their distinct, non-overlapping membrane placement. Membrane affinity and the achieved spatial arrangement are vital in creating signal transduction.

**Fig. 4:**
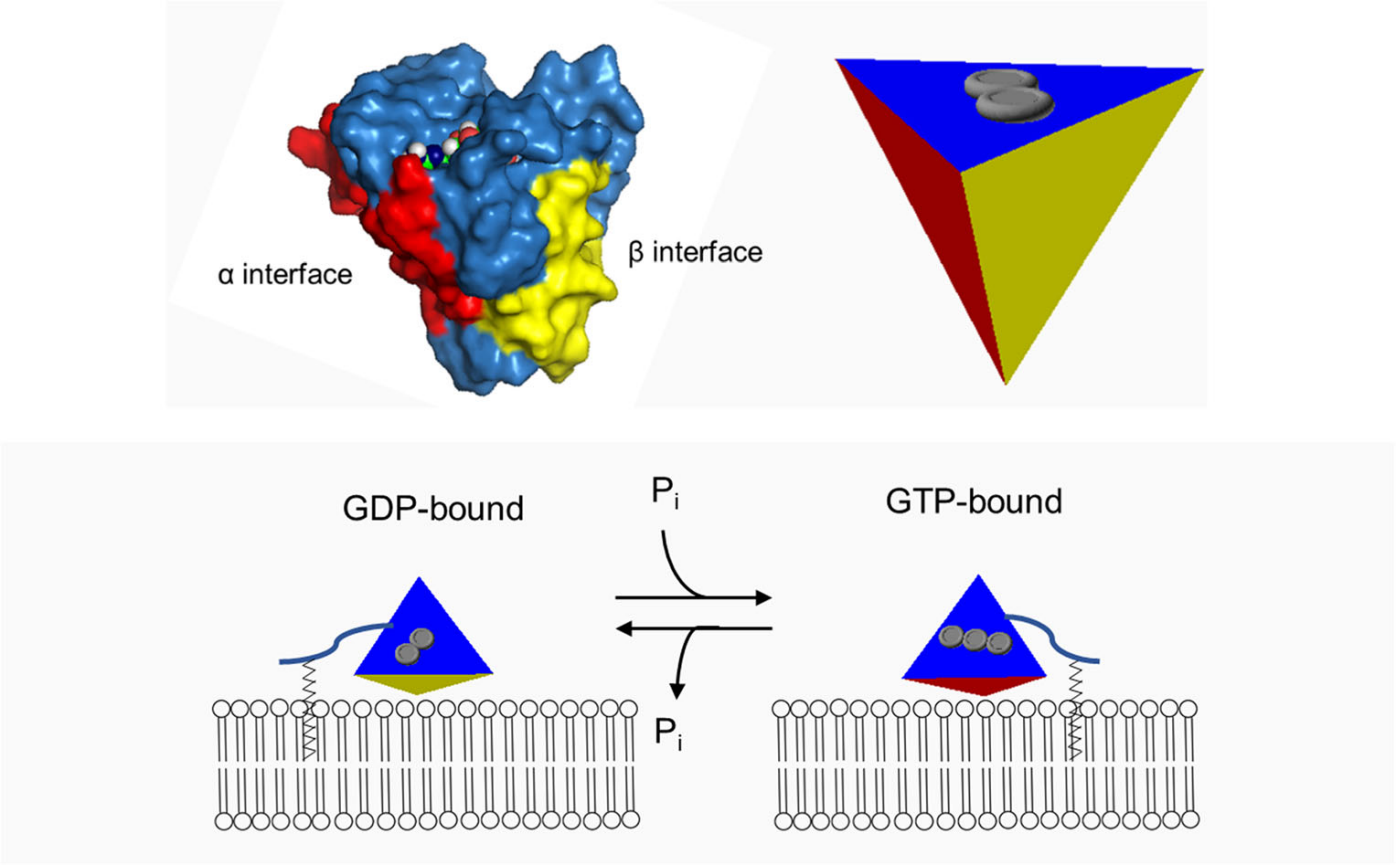
Membrane-association modes of Ras proteins. In GDP-bound form, β interface is preferred (formed by β1, β2, and β3, yellow colored), while in GTP-bound form, the protein floats to the membrane on the α interface (L3, α4, β6 and α5, red colored) [73]. The schematic protein is shown above the scheme next to the 3D structure (surface) which was produced by using The PyMOL Molecular Graphics System, version 2.0 Schrödinger, LLC software, using PDB code: 3KY8 (H-Ras-GppNHp)

Medium throughput FRET measurements combined with MD simulations lead to the realization that the relative orientation of Ras with respect to the membrane is not only isoform- but also nucleotide-dependent, and the emerging orientation can display or hide the effector binding region of the protein, adding an extra layer to the complexity of the activation process. Certain residues of α4 and the HVR (Arg128, Arg135 and Arg169, Lys170, respectively) were shown to play a critical role in stabilizing the membrane-bound state, and L3 and α5—that were also shown to be allosterically coupled to the switch regions—were found to exert a nucleotide-dependent influence over them. The double Arg128Ala/Arg135Ala (in α4 helix) mutation was shown to impair signal output of GTP-loaded H-Ras-Gly12Val. Based on the findings, the following model for activation was proposed: GTP binding reorders switch I and switch II, and this causes the reorientation of β2 and β3 and the L3 loop connecting them, triggering coupled changes in α5, and this changes the conformation of the immediately adjacent basic residues (Arg169 and Arg170) inducing reorientation of α4 and the eventual tipping of the entire membrane-bound GTP-H-Ras, opening access to the hitherto shielded switch I loop: the effector binding site [71]. The notion that Ras proteins tilt at the membrane surface in a controlled and functionally relevant mode was reaffirmed by further MD simulations containing the full-length GTP-bound H-Ras [72] and the NMR-based structure of K-Ras in complex with a membrane-mimetic. The structures allowed the further refinement of the membrane-associating surfaces of K-Ras, differentiating two major sites: the α interface formed by L3, α4, β6 and α5, and the β interface formed by β1, β2, and β3. While the GDP-bound form prefers interaction with the membrane through the β interface, GTP binding promotes a switch to the α interface, liberating the effector-binding site [73] (Fig. 4). This latter mode of membrane association also leaves the most potent dimerization surface of Ras intact, as was seen in another MD study. The authors describe the surface formed by α3 and α4 as the most probable site of dimerization and report that disruption of key salt bridge (Lys101-Glu107) of this motif changes clustering within the membrane but does not abolish membrane binding [74]. Not all simulations predict identical surfaces as the membrane-binding site, but models topologically agree (N-terminal β stands and the C-terminal α helices), and it was shown that the energy barrier of reorientation of Ras between close lying states is low. It was also shown that oncogenic mutations affect membrane-binding, K-Ras-G12D samples the catalytically competent α interface–binding mode over the inactive β interface–driven mode much more so than K-Ras-Gln61His does. This could be so because membrane reorientation of Ras seems to be driven by intrinsic conformational dynamics rather than changes in protein-lipid interaction patterns [75]. This conclusion was challenged by another study, which suggested that membrane composition is a deciding factor in Ras membrane orientation [76]. The full catalytic apparatus was studied in MD simulation of a model system containing two GTP-bound farnesylated and methylated full-length K-Ras molecules in the form of a membrane-bound dimer, complexed with two effector molecules (a truncated model of Raf containing its membrane-binding and Ras-binding domains). The authors found that to allow both anchoring of Raf to the membrane and stable Raf-Ras binding, Ras has to assume and orientation that interacts with the membrane at the α3 and α4 helices. Simulations illustrate that membrane-bound Ras dimers can recruit Raf and promote its dimerization—thus create the growth signal.

Dynamics on fast time scale were compared for GDP-bound H-Ras(1-166), non-farnesylated H-Ras(1-185), and full-length farnesylated H-Ras(1-189) to investigate the effect of HVR and farnesylation on G domain using NMR measurements[32]. The C-terminal α5 helix extends to residue Asn172 in longer variant; the remaining 17 residues in HVR were unstructured. C-terminal truncation caused slight structural and dynamic perturbations that were propagated throughout the H-Ras protein: flexibility of the central β-sheet, residues in L2 loop preceding switch I, and α5 helix increased. The significant decrease of *R*_1_ values and increase of *R*_2_ values indicate slower molecular tumbling (larger *τ*_c_) suggesting the increase in the molecular size. Although the authors did not mention it, this could be an indication for HVR-induced dimerization of Ras-GDP. They found little effect of HVR farnesylation on the structure and dynamics of full-length H-Ras.

Clearly, findings concerning the membrane association of Ras proteins open new therapeutic potential: the isoform and mutational state–dependent oncogenic signaling can be inhibited by molecules which promote or stabilize membrane orientations that occlude the effector binding site.

### Dynamics in small-molecule binding

Proteins in general, but this is especially true for Ras proteins, exist in solution as the ensembles diverse conformers, from which interaction partners can select the one required for their optimum binding. According to the so-called conformational selection model, the binding event shifts the native conformational weights of the conformers toward that withdrawn from the ensemble by the bound state, as in any other chemical equilibrium [77]. Along the Ras catalytic cycle, besides the GDP-bound form, the state 1 conformation of the GTP-loaded state and conformations which block the effector-binding site at the membrane present possibilities that might lead to inhibition based on this conformational selection scenario.

State 1—the inactive GTP-bound form—came into spotlight again as another possible therapeutic target when an MD-derived, NMR-validated ensemble of this form was shown to possess a switch I–gated cavity [61]. A state 1-specific small molecule could be used to shift the conformational equilibrium of Ras-GTP toward the inactivated form inhibiting Ras-effector interaction—which can only take place when Ras-GTP is in state 2 conformation. This idea was used earlier too, and for this purpose, small molecules Zn^2+^–bis(2-picolyl)amine (Zn^2+^-BPA) and Zn^2+^-cyclen were tested and found to be effective in the case of not only wild-type H-Ras and K-Ras but also the Gly12Val oncogene mutants [33, 78].

Slow dynamics of GDP-bound H-Ras(1-171) was also investigated together with its Gly12Val oncogene mutant by relaxation dispersion measurements of bilinear coherences as well as classical CPMG experiments performed at 5 °C [30]. The authors found that the α1, α2, α3, L2, L4 loops, central β1–β3-sheet lining the binding pockets of previously described inhibitors for Ras, undergo large changes on slow time scale, suggesting the possibility of selective targeting of some of them. Intriguingly, they found some residues at the end of the α3 helix (104–107) as well as residue 122 did not show motions on this slow time scale even though they are involved in inhibitor binding, suggesting that these residues might have motions on a different time scale (probably faster) than what can be probed by these experiments. This assumption could be confirmed by the results of earlier protein-based fast time scale analysis conducted for GDP-bound H-Ras(1-166) [79] and H-Ras(1-171) [40]: residues 27–32, 58–66, and 107–109 (loops L2, L4, and L7, respectively) exhibited fast internal motions in GDP-bound form. This complex binding mechanism which involves selection from protein conformers of multiple time scale motions show a nice example for the interplay of conformational selection and induced fit binding. Notably, Lipari-Szabo analysis of GDP-bound K-Ras(1-169) with or without acetylation mimicking the K104Q mutant exhibited similar dynamical properties to H-Ras, where reduced *S*^2^ values were obtained in switch II, L7 (residues 104–109), 122–123 [80]. Intriguingly, only one difference was shown: switch I engaged in slower motions in case of K-Ras-GDP, which has not been detected in case of H-Ras previously.

## Conclusions

The classical biomolecular approach for understanding enzymatic function and the design of inhibitors/agonists rely on identifying robust structural changes, and the key residues responsible for such changes—and uses this information to understand and alter molecular systems. However, in case of Ras, the catalytic core of the molecule, the P-loop, and switch I and switch II regions, are all flexible, connected, and correlated to each other and also to the allosteric and membrane-binding regions. This integrated machinery involves the entire protein matrix providing the background of catalysis. Forming the primary port for interaction partners, the switch regions have unique conformations in the effector bound state, but also in the GEF- and GAP-bound forms, and are affected by dimerization and membrane binding, through crosstalk *via* hotspots linked by either H-bonds, hydrophobic interaction, or spatial closeness. Therefore, understanding the interaction networks that interlace the protein and the conformational freedom—susceptibility for change—of structural motifs is of prime importance. All these faces of K-Ras, however, are encoded in its structure and manifest through internal dynamics. Understanding the dynamics of these intricate molecular switches may open new frontiers of therapeutic significance. The mutation state and isoform-sensitivity of the dynamics and the populations of the sub-states present new, selective targets for drug design efforts, in diverse locations of the protein matrix hitherto not considered, distinct from the near-undruggable P-loop.

## Abbreviations

HVR: Hypervariable region of Ras
NMR: Nuclear magnetic resonance spectroscopy
EM: Electron microscopy
EGF: Epidermal growth factor
AFM: Atomic force microscopy
ECD: Electronic circular dichroism spectroscopy
VCD: Vibrational circular dichroism spectroscopy
IR: Infrared spectroscopy,
FRET: Förster-resonance energy transfer
DLS: Dynamic light scattering
SAXS: Small-angle X-ray scattering
*T*_1_ (= *R*_1_^−1^): Spin-matrix relaxation term measures the loss of the NMR signal intensity,
*T*_2_ (= *R*_2_^−1^): Spin-spin relaxation term manifests in terms of NMR signal’s broadening,
HetNOE: A steady-state ^1^H,^15^N heteronuclear NOE measured as the intensity (*I*_15N_) ratio of NOE_15N-1H_ = *I*_15N_ (H_irradiated_) / *I*^0^_15N_ (H_non-irradiated_)
*τ*_c_: The average time it takes for a molecule to rotate one radian, in the order of picoseconds for small and ns for larger molecules
RD: Relaxation dispersion (measurements)
RDC: Residual dipolar coupling
CPMG: Carr-Purcell-Meiboom-Gill sequence-based NMR experiment
CEST: Chemical exchange saturation transfer
DEST: Dark-state exchange saturation transfer
EXSY: Exchange spectroscopy
NOESY: Nuclear Overhauser effect-based spectroscopy
GDP: Guanosine-diphosphate
GTP: Guanosine-triphosphate
GppNHp: 5′-guanylyl-imidodiphosphate
GTPγS: Guanosine 5′-O-(γ-thio)-triphosphate
GppCH_2_p: Guanosine 5′-(β,γ-methylene)-diphosphate
DD: Dipole-dipole interaction
CSA: Chemical shift anisotropy
HSQC: Heteronuclear single-quantum coherence
MD: Molecular dynamics simulation
PDB: Protein Data Bank
QM/MM: Quantum mechanics/molecular mechanics simulation
wt: Wild type
